# Artificial neural network based prediction of postthrombolysis intracerebral hemorrhage and death

**DOI:** 10.1038/s41598-020-77546-5

**Published:** 2020-11-25

**Authors:** Chen-Chih Chung, Lung Chan, Oluwaseun Adebayo Bamodu, Chien-Tai Hong, Hung-Wen Chiu

**Affiliations:** 1 Clinical Big Data Research Center, Taipei Medical University Hospital, Taipei, Taiwan; 2 Department of Neurology, Taipei Medical University - Shuang Ho Hospital, New Taipei, Taiwan; 3 Department of Neurology, School of Medicine, College of Medicine, Taipei Medical University, Taipei, Taiwan; 4 Department of Hematology and Oncology, Cancer Center, Taipei Medical University - Shuang Ho Hospital, New Taipei, Taiwan; 5 Department of Medical Research and Education, Taipei Medical University - Shuang Ho Hospital, New Taipei, Taiwan; 6 Graduate Institute of Biomedical Informatics, College of Medical Science and Technology, Taipei Medical University, 250 Wu-Hsing Street, Sinyi District, Taipei City 110, Taiwan

**Keywords:** Machine learning, Predictive medicine, Neurology, Stroke

## Abstract

Despite the salient benefits of the intravenous tissue plasminogen activator (tPA), symptomatic intracerebral hemorrhage (sICH) remains a frequent complication and constitutes a major concern when treating acute ischemic stroke (AIS). This study explored the use of artificial neural network (ANN)-based models to predict sICH and 3-month mortality for patients with AIS receiving tPA. We developed ANN models based on evaluation of the predictive value of pre-treatment parameters associated with sICH and mortality in a cohort of 331 patients between 2009 and 2018. The ANN models were generated using eight clinical inputs and two outputs. The generalizability of the model was validated using fivefold cross-validation. The performance of each model was assessed according to the accuracy, precision, sensitivity, specificity, and area under the receiver operating characteristic curve (AUC). After adequate training, the ANN predictive model AUC for sICH was 0.941, with accuracy, sensitivity, and specificity of 91.0%, 85.7%, and 92.5%, respectively. The predictive model AUC for 3-month mortality was 0.976, with accuracy, sensitivity, and specificity of 95.2%, 94.4%, and 95.5%, respectively. The generated ANN-based models exhibited high predictive performance and reliability for predicting sICH and 3-month mortality after thrombolysis; thus, its clinical application to assist decision-making when administering tPA is envisaged.

## Introduction

Thrombolysis using intravenously administered recombinant tissue plasminogen activator (tPA) represents the standard of care and most effective treatment for acute ischemic stroke (AIS)^1,2^. However, the intended benefits of tPA is dampened by the risk of symptomatic intracerebral hemorrhage (sICH). Post-thrombolysis sICH herald poor clinical outcome and account for most early excess deaths^1^, with a high 3-month mortality rate^3^. This necessitates the development of therapy-decision support tools based on the thrombolysis risk-to-benefit ratio, especially as the accurate and early identification of patients with high risk of post-tPA sICH and death is increasingly shown to play a crucial role in informing clinicians’ decision-making and therapeutic strategies.

In the last decade, several scoring tools have been introduced to predict sICH or death after tPA^4–9^, however, the related studies only demonstrate moderate prediction accuracy and this has not translated into reduced incidence of sICH or increased 3-month clinical outcome. This, in part, forms the basis of the present study which explored the use of artificial neural network (ANN)-based predictive models for identifying patients with high risk of post-thrombolysis sICH and death, as well as stratification based on likelihood of benefiting from tPA therapy.

Evolving medical adaptation of artificial intelligence (AI) proffers the means to investigate nonlinear data relationships, enhance data interpretation, and design more efficient diagnostic and predictive tool^10^. ANN, an AI method simulating the structure and functionalities of the human neural architecture, is able to predict existent complex relationships between input and output variables by repeated learning and validation process, and is increasingly applied in various aspects of medical diagnosis and prediction^11,12^. The present study generated highly reliable ANN computational models to predict the risk of (i) sICH, and (ii) 3-month mortality amongst patients with AIS after treatment with intravenous tPA.

## Materials and methods

### Participants

Medical records of patients diagnosed with and/or treated for AIS between January 2009 and December 2018 were retrieved from Taiwan Stroke Registry^13^ of Shuang Ho Hospital, and retrospectively reviewed. The inclusion criteria were as follows: patients with AIS aged 18 or older, who received intravenous tPA treatment consistent with the updated American Heart Association/American Stroke Association guidelines^2^. The exclusion criteria were as follows: initial National Institute of Health Stroke Scale (NIHSS) score of < 4 or > 25, intra-arterial thrombectomy or other endovascular intervention for AIS. All patients underwent non-contrast head computed tomography (CT) or brain magnetic resonance imaging (MRI) before tPA. The Glasgow Coma Scale (GCS) was used to evaluate the baseline consciousness level of the patients. Fasting glucose, glycated hemoglobin (HbA1c), triglyceride, and low-density lipoprotein (LDL) levels were measured within 72 h after tPA. A repeated head CT or MRI was performed within 72 h of admission. SICH was defined as CT or MRI scan-documented bleeding from any part of the brain, associated with investigator-adjudged clinical deterioration, or presence of adverse events indicating clinical exacerbation within 72 h after tPA^14^. All CT/MRI results were analyzed by two independent neurologists and a radiologist. NIHSS scores, and 3-month modified Rankin Scale (mRS) scores were assessed by certified stroke specialists. In-hospital death and 3-month mortality were defined by a mRS score of 6 at discharge and during follow-up period of 3 months, respectively.

### Ethical approval

The study was approved by the Joint Institutional Review Board of Taipei Medical University (TMU-JIRB Approval No. N201705044). Waivers of informed consent were approved by the TMU-JIRB for this retrospective study involving the secondary analysis of existing data. All methods were performed in accordance with the relevant guidelines and regulations.

### Statistical analyses

All statistical analyses were performed using the JMP software, version 11.0.0 (SAS Institute Inc., Cary, NC, USA). Variables were summarized using descriptive statistics. Continuous variables with normal distribution are presented as mean ± standard deviation, and categorical variables are expressed as percentages with corresponding 95% confidence intervals (CIs). One-way ANOVA was used for continuous variables, and Fisher’s exact test was used for categorical variables. A *p*-value of < 0.05 was considered statistically significant.

### Application of ANN modeling

**Figure 1:**
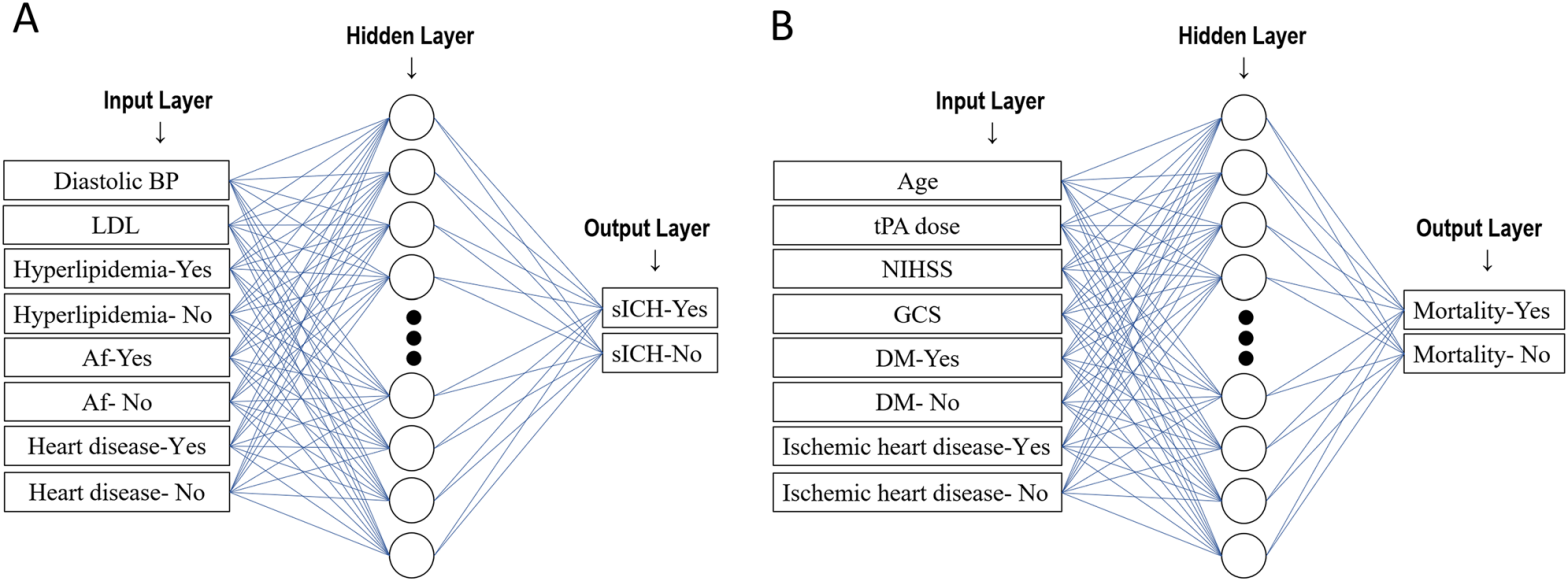
Artificial neural network (ANN) models in the present study. Schema showing the input, hidden, and output layers of (**A**) ANN model 1 to predict sICH, and (**B**) ANN model 2 to predict the 3-month mortality. Af, atrial fibrillation; ANN, artificial neural network; BP, blood pressure; DM, diabetes mellitus; GCS, Glasgow Coma Scale; sICH, symptomatic intracranial hemorrhage; LDL, low-density lipoprotein; NIHSS, National Institutes of Health Stroke Scale.

All ANN models were developed using STATISTICA 10.0 (StatSoft, Tulsa, Oklahoma, USA). The applied computational architecture was multilayer perceptron (MLP), a feed-forward ANN, combined with back-propagation algorithm for training the feed-forward ANNs. To train the ANN, supervised learning was performed by providing a series of input and output variables from the training dataset, such that by iteratively adjusting the connection weights, a desirable input–output mapping function was generated^12^. After appropriate training, optimization of the ANN architecture is initiated until the most satisfactory performance is achieved. The number of neurons in the hidden layer is set empirically. For our models, the input layer consisted of 8 neurons and the output layer consisted of 2 neurons (Figs. 1A,B).

### Model development

The input attributes of the ANN models included clinical features extracted from the patients’ medical history that were associated with the output attributes of interest, namely post-tPA sICH and 3-month mortality for model 1 and model 2, respectively. The generalizability of the analysis was assessed by fivefold cross-validation. Briefly, for cross-validation, the dataset was randomly shuffled and partitioned into five subsets (folds), followed by five rounds of training and validation of the ANN models. In each round of the analysis, four subsets served as the training subsets, and the remaining 1 subset was retained to validate the ANN model. Each of the five subsets was only used once as the validation set in the cross-validation process.

The performance of the ANN models was evaluated using the five independent validation sets. The model performance was measured and visualized by the area under the receiver operating characteristic curve (AUC) of the training and validation sets, and the mean accuracy, precision, sensitivity, and specificity of the five validation sets were also reported.

## Results

### Cohort demographical and baseline clinicopathological characteristics

During the study period, 380 patients received tPA treatment for AIS. Among them, 331 patients (133 women and 198 men, mean age 69.2 ± 12.2 years) were eligible and enrolled into this study. Forty-nine patients who received endovascular interventions following intravenous tPA were excluded from baseline analyses. The average onset-to-treatment time was 122.1 ± 45.3 min. The mean baseline NIHSS score was 12.6 ± 6.3. The stroke subtypes of the cohort included large artery atherosclerosis (41.0%), cardioembolism (22.9%), small vessel occlusion (25.0%), and others (11.1%). There were 68 patients (20.5%) aged over 80 years. At baseline, older age was associated with lower GCS scores (*p* < 0.0001), higher initial NIHSS scores (*p* < 0.0001), lower body weight (*p* < 0.0001) and lower total tPA dose (*p* < 0.0001).

### Clinical outcomes after thrombolytic therapy

**Figure 2:**
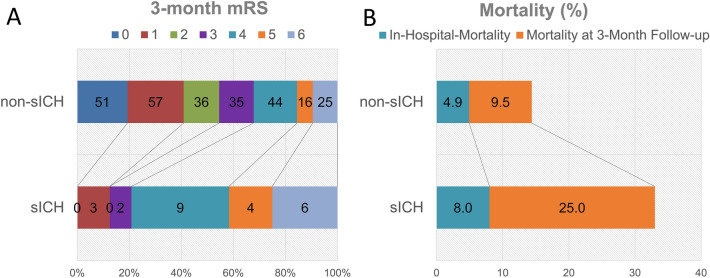
The 3-month functional outcomes of the patients with and without sICH after tPA treatment. Chart showing the differential 3-month mRS of patients with or without sICH of tPA administration (**A**). The numbers in each color-coded bar indicate the number of patients from the sICH or non-sICH group. (**B**) Graphical representation of the mortality outcomes of patients with and without sICH during hospitalization and during the 3-month post-tPA follow-up period. Numbers in the color-coded bar represent the percentage of patients in each group. sICH, symptomatic intracranial hemorrhage; mRS, modified Rankin Scale; tPA, tissue plasminogen activator.

Within 72 h of administering tPA, 25 patients (7.6%) exhibited sICH (Table 1). Among these, 2 patients (8.0%) died during hospitalization. During the 3-month follow-up after tPA, 43 patients (~ 13.0%) from the total cohort were lost to follow-up, and the remaining 288 patients were enrolled into the ANN analysis for post-tPA 3-month mortality (Table 2). Of these 288 patients, 31 deaths were recorded (17 in-hospital and 16 after discharge). Compared with the non-sICH group, patients who developed sICH after tPA had less favorable outcomes, as demonstrated by higher mRS scores (Fig. 2A), and greater risk of 3-month mortality (Fig. 2B) during the first 3 months post-tPA.

**Table 1:** Baseline characteristics of patients with and without symptomatic intracranial hemorrhage (sICH) after tPA.

	sICH	Non-sICH	*p*-value
n	25	306	
Age	68.1 ± 11.0	69.2 ± 12.3	0.62
Female, n (%)	14 (56)	119 (38.9)	0.09
Onset-to-hospital time, minutes	49.8 ± 36.2	61.0 ± 44.9	0.16
Onset-to-treatment time, minutes	118.0 ± 45.9	122.5 ± 45.3	0.64
BMI, kg/m^2^	24.7 ± 5.0	24.9 ± 4.0	0.87
tPA total dose, mg	54.6 ± 15.2	55.9 ± 13.8	0.71
Glasgow coma scale score	11.6 ± 4.2	11.8 ± 4.5	0.88
Systolic BP (mmHg)	163.9 ± 22.2	160.1 ± 29.9	0.45
Diastolic BP (mmHg)	100.5 ± 14.7	90.4 ± 19.2	0.004*
Baseline NIHSS	14.0 ± 5.7	12.4 ± 6.4	0.20
**Stroke subtype, n (%)**			
Cardioembolism	8 (33.3)	58 (22.0)	0.046*
Large-artery atherosclerosis	10 (41.7)	108 (40.9)	
Small-vessel occlusion	1 (4.2)	71 (26.9)	
Others	5 (20.8)	27 (10.2)	
**Risk factors, n (%)**			
Hypertension	22 (88)	219 (72.8)	0.10
Diabetes mellitus	10 (40)	95 (31.6)	0.39
Hyperlipidemia	3 (12)	92 (30.6)	0.05*
Atrial fibrillation	15 (60)	82 (27.4)	0.001*
Previous stroke or TIA	5 (20)	55 (18.3)	0.83
Heart disease	16 (66.7)	90 (31.4)	0.0005*
Smoking	3 (12)	63 (20.9)	0.29
**Laboratory data**			
Glucose at admission (mg/dL)	155.0 ± 55.5	154.2 ± 65.0	0.95
Glucose, fasting (mg/dL)	149.0 ± 71.1	132.0 ± 46.3	0.27
Creatinine (mg/dL)	1.1 ± 0.7	1.2 ± 1.1	0.54
Cholesterol (mg/dL)	174.0 ± 42.3	187.5 ± 42.9	0.14
Triglyceride (mg/dL)	112.5 ± 65.0	127.0 ± 89.8	0.32
LDL (mg/dL)	100.1 ± 30.2	114.2 ± 36.0	0.035*
HbA1c (%)	6.4 ± 1.3	6.4 ± 1.4	0.93
**Outcome measures**			
In-hospital death n (%)	2 (8)	15 (4.9)	0.53
Death at 3-month, n (%)	6 (25)	25 (9.5)	0.02*
3-month mRS	4.2 ± 1.6	2.4 ± 1.9	< 0.0001*

Continuous variables are presented as mean ± SD. One-way ANOVA was used for continuous variables, and Fisher’s exact test was used for categorical variables. BMI, body mass index. BP, blood pressure. HbA1c, hemoglobin A1c. sICH, symptomatic intracranial hemorrhage. LDL, low-density lipoprotein. NIHSS, National Institutes of Health Stroke Scale. mRS, modified Rankin Scale. TIA, transient ischemic attack. tPA, tissue plasminogen activator. *p-value < 0.05.

**Table 2:** Comparison of clinical variables between dead and surviving patients with acute ischemic stroke at 3 months post-tPA.

	Dead	Survivors	*p*-value
n	31	257	
Age	75.5 ± 12.6	68.7 ± 11.8	0.0067*
Female, n (%)	16 (51.6)	104 (40.5)	0.23
Onset-to-hospital time, minutes	51.6 ± 41.0	61.8 ± 44.7	0.21
Onset-to-treatment time, minutes	110.8 ± 39.4	123.0 ± 45.2	0.12
BMI, kg/m^2^	24.4 ± 3.9	25.0 ± 4.2	0.41
tPA total dose, mg	49.0 ± 13.2	56.3 ± 14.0	0.008*
Glasgow coma scale score	9.5 ± 5.2	12.1 ± 4.4	0.015*
Systolic BP (mmHg)	156.5 ± 34.4	160.4 ± 29.2	0.59
Diastolic BP (mmHg)	87.1 ± 19.7	91.6 ± 19.7	0.29
Baseline NIHSS	18.4 ± 5.9	11.8 ± 5.9	< 0.0001*
**Stroke subtype, n (%)**			
Cardioembolism	5 (21.7)	55 (23.4)	0.07
Large-artery atherosclerosis	14 (60.9)	95 (40.4)	
Small-vessel occlusion	1 (4.4)	65 (27.7)	
Others	3 (13.0)	20 (8.5)	
**Risk factors, n (%)**			
Hypertension	23 (79.3)	188 (73.2)	0.47
Diabetes mellitus	14 (48.3)	75 (29.2)	0.035*
Hyperlipidemia	5 (17.2)	75 (29.2)	0.17
Atrial fibrillation	7 (24.1)	81 (31.6)	0.41
Previous stroke or TIA	7 (24.1)	43 (16.7)	0.32
Ischemic heart disease	10 (35.7)	30 (12.2)	0.0008*
Smoking	4 (13.6)	52 (20.2)	0.41
**Laboratory data**			
Glucose at admission (mg/dL)	177.8 ± 96.4	150.3 ± 58.9	0.18
Glucose, fasting (mg/dL)	181.0 ± 77.4	128.8 ± 42.0	0.005*
Creatinine (mg/dL)	1.2 ± 1.0	1.8 ± 1.7	0.08
Cholesterol (mg/dL)	179.3 ± 36.6	186.5 ± 43.6	0.37
Triglyceride (mg/dL)	107.1 ± 80.2	126.5 ± 85.2	0.28
LDL (mg/dL)	106.8 ± 28.4	112.8 ± 36.3	0.34
HbA1c (%)	6.6 ± 1.5	6.4 ± 1.4	0.62
**Pre-discharge condition, n (%)**			
sICH	6 (19.4)	18 (7.0)	0.02*
In-hospital death	17 (54.8)	0 (0)	< 0.0001*

Continuous variables are presented as mean ± SD. One-way ANOVA was used for continuous variables, and Fisher’s exact test was used for categorical variables. BMI, body mass index. BP, blood pressure. HbA1c, hemoglobin A1c. sICH, symptomatic intracranial hemorrhage. LDL, low-density lipoprotein. NIHSS, National Institutes of Health Stroke Scale. mRS, modified Rankin Scale. TIA, transient ischemic attack. tPA, tissue plasminogen activator. *p-value < 0.05.

### Patients with sICH presented with an exacerbated clinical phenotype compared with the non-sICH group

The demographics and baseline clinicopathological characteristics of patients with or without post-tPA sICH are presented in Table 1. At baseline, patients with sICH exhibited higher diastolic BP, lower LDL level, and higher prevalence of atrial fibrillation or any other type of heart disease, lower prevalence of hyperlipidemia, when compared with patients who did not develop sICH. The sICH group also differed in their stroke subtypes to the non-sICH group. There were more patients with the cardio-embolic subtype and fewer small-vessel occlusions in the sICH group.

### Attributes associated with mortality within 3 months of thrombolytic therapy

The demographics and mortality-related clinicopathological characteristics of our total cohort in the first 3 months after tPA are presented in Table 2. We observed that patients who were older, received lower dose of tPA, had lower GCS and higher NIHSS score at baseline, co-morbid with diabetes mellitus (DM) and ischemic heart disease, and higher fasting glucose level, were more likely to die within the first 3 months after tPA therapy.

### Random oversampling

Understanding the detrimental effect of an imbalance or severely skewed dataset on the accuracy and generalizability of any prediction model, we sought to reduce the disproportionate ratio of our sICH to non-sICH and the 3-month mortality patients to survivors in the cohort, and performed random oversampling of the minority classes, namely the sICH and 3-month mortality subsets. Thus, one hundred samples were randomly selected from the sICH or 3-month mortality subset to increase the size of the training and validation sets and rebalance the class distribution for the ANN model 1 or model 2, respectively. This naïve method not only rebalances the class distribution, but also improve overall classification performance^15,16^. After random oversampling, 100 sICH and 306 non-sICH samples were randomly partitioned into 326 training and 80 validation sets in ANN model 1, and 100 mortality samples and 257 survivals were randomly partitioned into 285 training and 72 validation sets in ANN model 2, respectively (Table 3).

**Table 3:** Description of the input attributes within the training and validation datasets of the ANN models.

ANN model 1			
	Training (n = 326)	Validation (n = 80)	Total (n = 406)
Diastolic BP (mmHg)	92.7 ± 0.4	92.8 ± 1.5	92.7
LDL (mg/dL)	110.2 ± 1.3	110.2 ± 5.0	110.1
**Hyperlipidemia**			
Yes, %	25.4 ± 1.9	25.5 ± 7.6	25.4
No, %	74.6 ± 1.9	74.5 ± 7.6	74.6
**Atrial fibrillation**			
Yes, %	34.8 ± 0.9	34.8 ± 3.6	34.8
No, %	65.2 ± 0.9	65.2 ± 3.6	65.2
**Heart disease**			
Yes, %	38.9 ± 0.6	38.9 ± 2.3	38.9
No, %	61.1 ± 0.6	61.1 ± 2.3	61.1

ANN model 2			
	Training (n = 285)	Validation (n = 72)	Total (n = 357)
Age (years)	70.7 ± 0.3	70.5 ± 0.8	70.5
tPA total dose (mg)	54.7 ± 0.3	54.7 ± 1.3	54.7
Baseline NIHSS	13.6 ± 0.1	13.6 ± 0.5	13.6
Glasgow coma scale	11.3 ± 0.2	11.3 ± 0.7	11.3
**Diabetes mellitus**			
Yes, %	34.5 ± 1.0	34.4 ± 4.1	34.5
No, %	65.5 ± 1.0	65.6 ± 4.1	65.5
**Ischemic heart disease**			
Yes, %	18.4 ± 0.7	18.4 ± 2.8	18.4
No, %	81.6 ± 0.7	81.6 ± 2.8	81.6

Continuous input attributes are presented as the mean values of the attributes and the standard deviation within the five training and validation sets. Categorical input attributes are presented as the percentage of the mean case numbers and standard deviation within the five training and validation sets. ANN, artificial neural network. BP, blood pressure. LDL, low-density lipoprotein. NIHSS, National Institutes of Health Stroke Scale. tPA, tissue plasminogen activator.

### Predictive performance of our post-tPA sICH and 3-month mortality ANN models

#### ANN model 1

**Figure 3:**
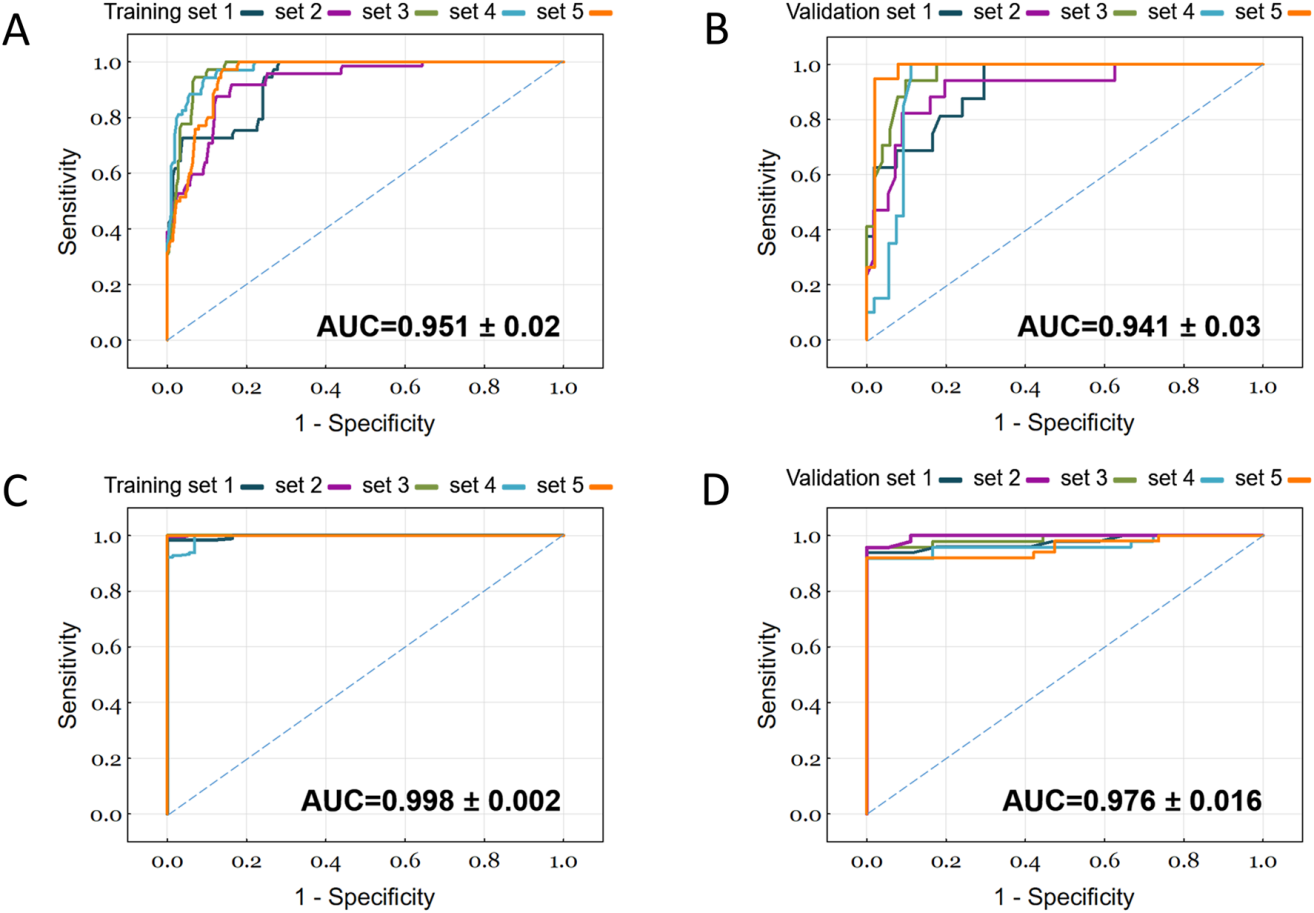
Predictive performance of the ANN models. Visualization of the ROC curves and AUC of the five (**A**) training and (**B**) validation sets used in the ANN model 1 to predict the post-tPA sICH of patients with AIS. Visualization of the ROC curves and AUC of the five (**C**) training and (**D**) validation sets used in the ANN model 2 to predict the post-tPA 3-month mortality of patients with AIS. The AUC value represents the mean ± SD of the AUC of the five training and validation sets. ANN, artificial neural network; AUC, area under the receiver operating characteristic curve; ROC, receiver operating characteristic; tPA, tissue plasminogen activator.

For model 1, the ANN was trained to predict post-tPA sICH. The input attributes, based on results of analyses in Tables 1, included the baseline diastolic BP, level of LDC, history of hyperlipidemia, Af, or any kind of heart disease (Table 3); however, while stroke subtype is associated with sICH, it was not included in the model because the accurate classification of stroke subtype would usually require a complete evaluation of stroke etiology and this was not obtainable before tPA treatment. The output attribute of this model was sICH. After adequate training, the ANN models that contained 8, 11, 15, 16, and 20 neurons in the hidden layer achieved the best predictive performance for the five validation sets (validation 1–5), with a mean training accuracy of 89.2 ± 4.1% and validation accuracy of 91.0 ± 3.5%. The mean precision of the validation sets was 81.0 ± 11.1%, sensitivity was 85.7 ± 14.0%, and specificity was 92.5 ± 5.4%. The mean AUC was 0.951 ± 0.02 for the training sets and 0.941 ± 0.03 for the validation sets (Figs. 3A,B).

#### ANN model 2

For model 2, the ANN was trained to predict the 3-month mortality following tPA. The input attributes include age, dose of tPA, baseline NIHSS score, GCS score, history of DM, and history of ischemic heart disease (Table 3). Fasting glucose level was not included in the model because this data was not available before the tPA therapy. After adequate training, the ANN models that contained 13, 15, 15, 15, and 20 neurons in the hidden layer achieved the best predictive performance for the five validation sets (validation 1–5), with a mean training accuracy of 99.3 ± 0.8% and a validation accuracy of 95.2 ± 1.9%. The mean precision of the validation sets was 89.2 ± 6.7%, sensitivity was 94.4 ± 7.9%, and specificity was 95.5 ± 3.0%. The mean AUC was 0.998 ± 0.002 for the training sets and 0.976 ± 0.016 for the validation sets (Figs. 3C,D).

### Comparison of the predictive performance of ANN models with other prediction scores

To better understand the predictive performance of ANN models and available outcome prediction scores, we calculated the Stroke Prognostication using Age and NIH Stroke Scale index (SPAN-100)^6^, Totaled Health Risks in Vascular Events (THRIVE)^7,17^, and Safe Implementation of Treatments in Stroke (SITS)^5^ scores with the present clinical data, and used receiver operating characteristic (ROC) curve analysis to compare our ANN models with the scores (Table 4). ANN model 1 and model 2 showed remarkably higher AUC values in ROC analysis than the scoring systems in predicting sICH and 3-month mortality, indicating the greater discrimination ability of the ANN models for the measured outcome.

**Table 4:** Comparison of the predictive performance of different models.

AUC value	Prediction of post-tPA sICH	Prediction of 3-month mortality
ANN	0.941	0.976
SPAN-100	0.511	0.754
THRIVE	0.621	0.789
SITS	0.648	0.728

The AUC value of ANN models showed the mean AUC of the five validation sets. The AUC value of SPAN-100 index was calculated using a univariable regression model with SPAN-100 score (age plus NIHSS). Compared to the SPAN, THRIVE, and SITS scores, the predictive performance was remarkably higher in the ANN models. ANN, artifice al neural network. AUC, the area under the receiver operating characteristic curve. NIHSS, National Institutes of Health Stroke Scale. sICH, symptomatic intracerebral hemorrhage. SITS, Safe Implementation of Treatments in Stroke score. SPAN-100, Stroke Prognostication using Age and NIH Stroke Scale index. THRIVE, Totaled Health Risks in Vascular Events score. tPA, tissue plasminogen activator.

## Discussion

The present study generated ANN-based predictive models to predict sICH within 72 h of intravenous tPA administration (model 1) and the post-tPA 3-month mortality (model 2) of patients with AIS. ANN model 1 and ANN model 2 achieved high validation performance, with AUC 0.941 and 0.976, respectively. This is predictive relevance, as the AUC measures and portrays the degree of separability; thus, the relatively high AUC values indicate that the models are capable of distinguishing the classes of interest, namely, sICH *versus* non-sICH and mortality *versus* survival of tPA administration. Our result demonstrated that high baseline diastolic BP, lower level of LDL, history of hyperlipidemia, Af, and heart disease were associated with sICH, while aging, lower dose of tPA, lower baseline GCS score, higher NIHSS score, history of DM and ischemic heart disease, were predictors of the post-tPA 3-month mortality. Thus, the rationale for the application of these variables as input attributes, and in part informs the demonstrated reliability and accuracy of our ANN-based predictive models.

Stroke is a multi-factorial neurological disorder with broad systemic implications. Individual factors/variables have been shown to be modestly associated with therapeutic outcome, and accurate prediction of clinical course or prognostication of clinical outcome would be difficult if based on a single clinical factor^18^. Thus, the complexity of AIS limits the conventional prediction models and scoring systems, as well as curtails their predictive reliability for the individualization of treatment. Against this background, in an effort to correctly predict which patients are at the greatest risk of post-thrombolysis sICH and death, the present study employed a composite and integrated prediction model consisting of multiple demographic and clinical factors, with the evidence-based predictive or prognostic capability in AIS. The complex synergy between these variables, we believe, is crucial for clinical application.

Consistent with contemporary knowledge, we identified several factors related to post-tPA sICH and 3-month mortality, and most of these factors are of demonstrable prognostic relevance in AIS. Underscoring the rationality of our selected input attributes, for example, high NIHSS score, and Af have been suggested to increase the risk of post-thrombolysis hemorrhagic transformation and poor AIS outcome^19,20^; in fact, Whiteley WN, et al. in a systematic review and meta-analysis of 55 studies showed that a higher stroke severity with an odds ratio of ~ 1.1 per NIHSS point is associated with post-tPA sICH, and that the odds double in the presence of Af-related cardioembolic stroke subtype^18^.

In addition, consistent with our finding that AIS patients at high risk for sICH were concurrently morbid with heart disease, there is indication that being comorbid with congestive heart failure or ischemic heart disease increases the risk of sICH after tPA in patients with AIS^18^. Evidence of brain–heart interactions continues to accrue, with cardiac dysfunction being associated with brain injury; concordant with the conclusions of a population-based 30-year cohort study, “Heart failure was associated with increased short-term and long-term risk of all stroke subtypes”^21^.

In our model 1, high diastolic BP was an indicator of sICH. This is corroborated by findings indicating that elevated BP within the first 24 h following tPA administration in patients with AIS is an independent predictor of sICH^22^, and that extremely high or low systolic and diastolic BP are significantly associated with mortality and disability^23,24^. Thus, guiding medical decision-making, to maintain optimal control of BP during the acute phase of AIS would play a therapeutically significant role in lowering the potential risks of sICH and improve clinical outcome^2,23^.

It has been suggested that low LDL level damages the integrity of the smooth muscle cells, impairs the endothelial function of cerebral vessels, and consequently increase the risk of hemorrhage^25^. The actual effect of altered serum lipid level on sICH might be confounded by the presence of Af^26^, as lower blood lipid levels have been shown in patients with Af, and hypolipoproteinemia has been touted to increase susceptibility to developing Af^26^. This is corroborated by our finding that LDL and hyperlipidemia were significantly associated with sICH in our AIS cohort, and informed their inclusion into the ANN model for predicting sICH before tPA treatment.

Aging has a negative effect on the long-term outcome of AIS, especially as patients aged > 80 years present with more severe AIS than their younger counterparts^27^, and this is consistent with higher NIHSS score being strongly linked with AIS-related mortality^8,28^. This is further corroborated by findings of the SPAN-100 index study which demonstrated the prognostic relevance of combining patients’ age (years) and baseline NIHSS score in patients with AIS^6^. In partial concordance, in our present study both patients’ age and NIHSS score were shown to be critical indicators, and were applied in the ANN model for predicting the post-tPA 3-month mortality for patients with AIS.

Furthermore, it is clinically relevant that in our AIS cohort, the patients who died within 3 months of intravenous thrombolysis (i.e. 3-month mortality group) received lower dose of tPA than the survivors. Intuitively, older patients had lower body weights and received lower total dose of tPA. In addition, 51.5% of our AIS cohort who were patients aged > 80 years received low dose tPA (0.6 mg/kg). This ‘aging’ concept may explain in part the strong association between lower tPA dose and the post-tPA 3-month mortality; this is more likely so, considering cumulative evidence of the safety, efficacy and therapeutic non-inferiority of low dose tPA, compared to standard dose^29–31^. In our model, the predictive value for tPA dose suggests the applicability of our ANN model to optimize the tPA dose for individual patients.

In our ANN model 2, DM was considered an essential input to predict mortality after tPA. This derives from statistical inference from our clinicopathological analysis, and consistent with published evidence that patients comorbid with DM have less favorable clinical outcomes, including higher death rate and more long-term morbidity after tPA for AIS^32^. Similarly, consistent with our observation that survivors had an lower initial GCS score in contrast to non-survivors, we considered the initial GCS score as a predictor of mortality after tPA for patients with AIS, and included it as one of the attributes of our ANN predictive model for post-tPA 3-month mortality. This is congruous with the findings of previous studies indicating that an impaired consciousness level is an early and independent indicator of unfavorable therapeutic outcome and mortality in patients with AIS^8,33^.

As already alluded, there are a number of studies focused on the prediction of sICH^4–7^ and death^7–9^ after tPA in patients with AIS. It is noteworthy that the SPAN-100 index which touted to be a simple and fast scoring system achieved a post-tPA sICH detection rate of only 42% in SPAN-100^pos^ patients, and data on the predictive power of this index are inconsistent^6,9^. Furthermore, the THRIVE score for predicting clinical outcome after thrombolysis which exhibited some superiority to other scoring systems, predicted the risk of hemorrhage and death with AUC of 0.64 and 0.72, respectively^7,34^. Similarly, the SITS score to predict the risk of sICH had an AUC of 0.70^5^. It is thus clinically-relevant that while these studies predict the sICH and mortality after tPA with moderate predictive power, our ANN predictive model AUC for sICH was 0.941, and the ANN predictive model AUC for 3-month mortality was 0.976, respectively.

Consistent with contemporary knowledge, our study demonstrates that the training of ANN under supervised learning could emulate human expert diagnostic performance and identify relevant predictive markers for any diagnostic task^12^. We herein exploited some of the benefits of neural networks, such as the requirement of little (or no) formal statistical training, minor user input, the implicit capacity to detect complex nonlinear relationships between dependent and independent variables, and the ability to delineate all possible interactions between predictor variables^35^. The high predictive power of our models is consistent with known application of ANN to assist with the diagnosis of stroke and prediction of its outcome^35–37^. More specifically, the present study demonstrated that the application of ANN helped improve the accuracy of predicting sICH within 72 h of intravenous tPA and the risk of death after the intravenous administration of tPA. For patients with high risk of post-tPA sICH and poor outcome, ANN may facilitate the timely institution of indicated adjunctive therapies, such as intra-arterial thrombolysis and mechanical thrombectomy to improve the patients’ quality of life and functional outcome. Thus, we demonstrate that ANN has the potential to assist clinicians in patient stratification, and to establish individualized treatment plans for optimized AIS management.

As with other studies of this sort, our study has some limitations. First, the sample size is relatively small and the study is based on single-center data. A larger cohort of more patients from a multi-center setting with variable characteristics may be needed for accurate representation of the disease population, and for derivation of robustly generalizable inferences. Secondly, the present study samples excluded patients who received intra-arterial thrombectomy or endovascular intervention, thus might have inadvertently excluded a patient bloc with poorer response to treatment with intravenous tPA and thereby underestimate the severity and poor outcomes of AIS in this cohort. Thirdly, the retrospective design of the present study using past registry data makes it almost impractical to rule out investigator bias in control selection. Further studies with a prospective design are therefore warranted to develop AI-based diagnostic and/or prognostic tools that can be continuously updated with new information and evolve with the improvement of medical knowledge and healthcare management. This will help to establish more accurate prediction tools and clinical decision support systems.

## Conclusion

Our study demonstrating that ANN techniques can predict sICH and 3-month mortality for patients with AIS who were treated with intravenous tPA, with high accuracy, does indicate that novel AI-based models can be used to derive new knowledge and improve current healthcare management. Results documented herein are potentially applicable in the emergent clinical setting of AIS and can aid decision-making for therapeutic plans.

## Data Availability

The datasets generated during and/or analysed during the current study are available from the corresponding author on reasonable request.
